# Development and validation of the Trust in Multidimensional Healthcare Systems Scale (TIMHSS)

**DOI:** 10.1186/s12939-024-02162-y

**Published:** 2024-05-08

**Authors:** Samantha B. Meyer, Patrick Brown, Michael Calnan, Paul R. Ward, Jerrica Little, Gustavo S. Betini, Christopher M. Perlman, Kathleen E. Burns, Eric Filice

**Affiliations:** 1 School of Public Health Sciences, University of Waterloo, 200 University Ave West, Waterloo, ON N2L 3G1 Canada; 2 Department of Sociology, University of Amsterdam, Amsterdam, The Netherlands; 3 School of Social Policy, Sociology and Social Research, University of Kent, Canterbury, UK; 4 Centre for Public Health, Equity & Human Flourishing, Torrens University, Adelaide, Australia; 5 Unity Health Toronto, Toronto, Canada

**Keywords:** Trust, Physicians, Quality indicators, health care, Health policy, Validation study

## Abstract

**Context:**

The COVID-19 pandemic has reignited a commitment from the health policy and health services research communities to rebuilding trust in healthcare and created a renewed appetite for measures of trust for system monitoring and evaluation. The aim of the present paper was to develop a multidimensional measure of trust in healthcare that: (1) Is responsive to the conceptual and methodological limitations of existing measures; (2) Can be used to identify systemic explanations for lower levels of trust in equity-deserving populations; (3) Can be used to design and evaluate interventions aiming to (re)build trust.

**Methods:**

We conducted a 2021 review of existing measures of trust in healthcare, 72 qualitative interviews (Aug-Dec 2021; oversampling for equity-deserving populations), an expert review consensus process (Oct 2021), and factor analyses and validation testing based on two waves of survey data (Nov 2021, *n* = 694; Jan-Feb 2022, *n* = 740 respectively).

**Findings:**

We present the Trust in Multidimensional Healthcare Systems Scale (TIMHSS); a 38-item correlated three-factor measure of trust in doctors, policies, and the system. Measurement of invariance tests suggest that the TIMHSS can also be reliably administered to diverse populations.

**Conclusions:**

This global measure of trust in healthcare can be used to measure trust over time at a population level, or used within specific subpopulations, to inform interventions to (re)build trust. It can also be used within a clinical setting to provide a stronger evidence base for associations between trust and therapeutic outcomes.

**Supplementary Information:**

The online version contains supplementary material available at 10.1186/s12939-024-02162-y.

## Introduction

There is no shortage of literature supporting the argument that public trust in healthcare providers and the health system promotes practices that contribute to the health of populations [1, 2]. The critical role of trust in health systems has never been more salient due to COVID-19, whereby health officials called upon the public to trust them as *the* authorities for information provision used to guide health related practices at a population level. To maintain this sense of authority – their legitimacy as an institution – public trust was critical [3]. However, public exposure to ‘alternative expertise’ increasingly challenges the authority of health systems and officials as *the* trusted source of information [4, 5]. For example, social media has drastically changed the way that individuals come to access and consume health information as these sites provide instant updates and rapid distribution of alternative information [6]. Trust is thus challenged when alternative forms of ‘expertise’ contradict information provided by healthcare providers – for example, as users increasingly see peers within their social media networks as authoritative and legitimate sources of information [7]. Further, research has demonstrated that the use of scientific terminology – e.g., evidence, science, research – signals that information is trustworthy, even if the sources of information are false or using such language to promote their own agenda [8]. As such, health officials – including providers and those guiding health system reform and health policy – are having to refocus their efforts on (re)building trust [9].

Trust, as something to be earned (to gain trust) or maintained (if one starts from a place of trust), is a valuable indicator of health system performance [10]. A reliable measure of trust, with demonstrated use in diverse populations, is thus critical for evaluating and informing interventions to improve trust, targeting doctors, health systems, and health policy. However, existing tools used to measure trust in healthcare have limitations in their ability to inform change at the doctor, system, and policy level through a single measure [11]. This limitation, among others detailed below, was instrumental in the conception of the present research. In the section that follows, we situate our work within the broader trust literature, and specifically the conceptualisation and measurement of trust in the context of healthcare. We then move on to discuss two empirical studies – one is the development (the ‘development study’), and the other is the validation (the ‘validation study’) – that led to the Trust in Multidimensional Healthcare System Scale (TIMHSS) – the first single measure that examines trust in the healthcare system broadly, including the doctor, system, and policy.

## Conceptual framework

Trust has be defined as “the mutual confidence that no party to an exchange will exploit the other’s vulnerability” [12] (p. 1133), with the trustor “accepting the risks associated with the type and depth of the interdependence inherent in a given relationship” [13] (p 422). While trust has been considered irrational [13], we argue that trust is a complex multidimensional concept consisting of both a rational component (arising from experience) and an non-rational component based on instinct and emotion [14].

Despite decades of research on trust in healthcare, there remains no widely accepted empirical conceptualisation and understanding of trust in health research [9]. Within health research, however, definitions of trust generally embody the notion of expectations by the public that healthcare providers and the health system will demonstrate competence, as well as act in the patient’s best interest [15, 16]. These expectations of providers are embedded within public perceptions and assumptions regarding broader systems of service organisation, professional expertise, and knowledge development [17, 18]. Trust, therefore, extends beyond relationships between patients and providers to include health systems and policy. Conceptually, the present work considers trust in healthcare as it relates to trust in both doctors and institutions, as well as the macro-level structures that govern their practice, recognising that trust occurs at two distinct levels: institutional and interpersonal [19, 20] – institutional trust is that which is placed in one or more social system or institution, while interpersonal trust is negotiated between individuals. Importantly, trust at these two levels does not operate independently. Trust placed in individuals to some extent impacts trust in the organisation they represent – or as Fulmer and Gelfand (2012) note, “Trust within any one level does not occur in a vacuum and needs to be considered in the context of trust and related factors at other levels” [21] (p. 1204). Scholars, however, remain in different minds regarding in who or what form of trust comes first [22, 23]. However, both the reputation and knowledge of the institution, and the personal relationships with those who represent it, are vital to the pursuit of trust [24]. Accounting for trust at these levels is critical for understanding where and how trust can be (re)developed and maintained and avoiding the mistake of investing resources in (re)building trust in an institution, for example, when those who represent it are not trusted.

Trust in the context of healthcare is difficult to research given that the conditions under which one makes health decisions vary considerably. For example, the decision to follow a doctor’s advice about a blood pressure tablet is very different than when one needs emergency surgery to remedy a heart condition – the former may be made after much consideration of risk and weighing of options, while the latter might involve little to no time to consider risk or alternatives. Research in healthcare has demonstrated that in some scenarios individuals may also *choose*, in a sense, to overlook potential failings of the healthcare system [25, 26] or *choose* to depend [27] as a strategy for minimizing anxiety and managing vulnerability. In a similar vein, research investigating trust in public hospitals found that some patients described situations of ‘forced or resigned trust’ in doctors. Because they wanted to trust, patients were found to be willing to except and sometimes excuse or justify the ‘problems’ of the health system in order to maintain their trust in providers [28]. The lack of perceived agency or choice in the latter example, however, calls to question whether patients are indeed trusting because trust is a reflexive choice – as such, we might consider their action to be one based on dependence or confidence over trust. The critical role of choice is evident, too, in cases where an individual does not consider if they trust until a point at which it is called into question. For example, instances whereby individuals adopt an ‘innocent until proven guilty’ approach, displaying little knowledge or interest in understanding, and thus considering trust, until the point at which the individual or institution is no longer considered trustworthy [29]. This latter point – the notion of trustworthiness as it relates to trust – is central to our work whereby “trust is a judgement by the trustor, requiring the acceptance of resultant vulnerability and risk, that the trustee (individual or organisation) has the competence, willingness, integrity and capacity (i.e., trustworthiness) to perform a specified task under particular conditions” [30] (p. 894). That is, our decision to trust (or not) is based on our assessment of whether the trustee has attributes worthy of trust. Trustworthiness, as Taylor writes “[places] the onus of responsibility on the entity looking to be trusted—most commonly, the clinician, the organization, or the system—to be worthy of trust.” As we present, within our measure of trust, respondents are asked to respond to how much they trust, in many cases by considering attributes of doctors and the health system as they demonstrate trustworthiness.

## Considerations in the measurement of trust

Trust, particularly in the context of healthcare, is difficult to measure given the inherent vulnerability of the patient [31]. As noted, patients may be forced to take risks where other options are non-existent, and the consequence of not taking risk may lead to a worse outcome [32]. Thus, when you ask a patient if they trust their healthcare provider, there are specific clinical contexts in which the ‘decision’ is more a ‘forced option’ [33] – to undergo a treatment or not. These subtle distinctions – the notion of choice and consideration of risk – are important in the measurement of trust and thus, it is critical in measurement development that related constructs are considered in the validation process. This also relates to the notable distinction between trust, distrust, and mistrust. They are semantically distinct concepts, associated with different attitudes and behaviours, and require separate scales and indices for measurement (see for example [34]). To not have trust does not suggest one distrusts [35]. While there are calls to seek convergence on the conceptual definitions of low trust, mistrust, and distrust [9], we focus specifically on the measurement of trust.

The conflation of trust with related yet distinct terms is critical to the development and validation of measures, yet this consideration has been overlooked in the creation of many existing measures. For example, despite research identifying semantic distinctions between trust and confidence [31, 36], measures continue to include confidence as a dimension of trust [11]. Further, research has demonstrated a conceptual distinction between trust and dependence, with the authors cautioning researchers who “may think they are measuring ‘trust’ and policy may be aimed at increasing ‘trust’, but unless both of them recognise the interplay between risk, familiarity and time, they may be measuring something other than trust” [27] (p. 13). Yet, no existing measures consider dependence for the purpose of discriminant validity. Trust has also been identified as distinct from, but correlated with, satisfaction, noting that the latter is an evaluation of previous experiences, while the former is primarily future-oriented [37] – as such, satisfaction might be considered for the purpose of convergent validity. The use of these constructs in the validation process is integral in our work.

Another notable challenge in measurement is differing perspectives on the dimensionality of trust [38] that we explore herein. While the view that trust is a multidimensional construct is prevalent, many empirical studies fail to differentiate between competence and other aspects of trust [37]. This may arise because of the difficulty individuals face in assessing a physician’s technical skills, or in differentiating between their technical and interpersonal skills. This may explain why, despite the recognised multidimensionality of the construct, of the 43 measures identified in a 2013 review, 60% were unidimensional [39]. A more recent systematic review of measures of trust in social institutions, including healthcare, identified benevolence, competence, and equity as the most recurring dimensions, but the category “other” was also one of the most frequent [11]. The authors argue that the growth of the ‘other’ category is not occurring due to the rise of new dimensions to reflect the evolution of trust but rather, is an inconsistency in taxonomy.

Central to our work is also the limited number of quantitative studies conducted to investigate trust in doctors, systems, and policy via a single measure [11]. Further, relative to other levels of analysis, literature on trust in health care organisations is small [9]. Within existing research, only two measures in healthcare look at doctors *and* macro-level structures; their results suggest that public trust in healthcare does comprise elements relating to institutional character [40, 41]. The need for a greater focus on the area of systems trust informs our approach to measure development.

Finally, since the development of popular measures of trust in healthcare (e.g., the Trust in Physician Scale [42]), there have been calls for researchers to assess the psychometric properties of measures of trust for diverse populations (e.g., racial and ethnic groups) [43]. Indeed, trust varies among subpopulations and is particularly critical for equity-deserving groups [44–47] whereby efforts to restore or build trust will require examination of the impact of racism and other forms of discrimination on the way the healthcare system treats patients [9]. The renewed commitment to (re)building trust should prioritize populations within which a lack of trust is leading to further disadvantage in terms of health outcomes. How and where to intervene can be facilitated by measures that account for dimensions most critical to diverse populations.

In the present work we respond to limitations of respected, yet dated measures, by interrogating the conceptualisation of the construct (dimensions and semantic distinctions that need to be made), and distinctions among the foci of trust (e.g., doctor vs. system vs. policy) among populations where trust is at greatest threat. We now present two studies – the ‘development study’ and the ‘validation study’ – that led to the construction of the Trust in Multidimensional Healthcare System Scale (TIMHSS).

## Development of the trust in multidimensional healthcare systems scale (TIMHSS)

### Methods for development study

The development study consisted of three separate phases: systematic review (item generation), qualitative interviews (item modification) and expert validation of items.

#### Phase 1 methods: systematic review (item generation)

In 2021 our team completed a review of existing scales used to measure patients’ trust (or distrust and mistrust) in the health system (including institutions and policies), healthcare providers or other entities related to the delivery of health services. Using the PRISMA approach for systematic reviews, we conducted a search in four databases and assessed a total of 26 articles. The psychometric properties of the scales were evaluated. Twelve new scales were identified, while 14 existing scales were adapted for different settings and populations. Further details of our methods to this review are presented elsewhere [11]. The review was conducted to identify gaps in existing measures since the publication of the 2012 review of measures of trust in healthcare conducted by Ozawa and Sripad. The review also provided conceptual content and empirical evidence foundational to version 1 (V1) item generation, the development of interview questions, and the interpretation of qualitative data.

#### Phase 2 methods: qualitative interviews (item modification)

Following Hall, Zheng, et al. [37], we conducted qualitative interviews with Canadians (*N* = 72) to modify/remove/create candidate items that emerged in interviews and were not already captured in V1. Quotas of sub-populations historically disadvantaged by social institutions were sampled with the goal of better understanding the dimensions of trust that might not be obtained with a representative or convenience sample: *n* = 7 First Nations, Métis and Inuit; *n* = 16 LGBT2SQ+; *n* = 8 low income (< $25,000CAD annual household income); *n* = 16 Black Canadians; *n* = 7 newcomers (less than 5 years living in Canada). An additional 18 participants were recruited who did not meet any criteria for the sampled equity-deserving populations.

Participants were recruited through Leger, Canada’s largest and most representative research marketing firm, to gain representation from harder-to-reach populations. Leger recruited potential participants and provided contact information to the research team. Recruitment of Black participants was also conducted via a WhatsApp group in Southern Ontario with members primarily of African decent, and through word of mouth. Interviews were conducted at three timepoints; 10 interviews were conducted with individuals identifying as LGBT2SQ+ in Feb-March 2019, 10 interviews were conducted with individuals identifying as Black in Oct-Nov 2020, and the remainder were conducted between Aug-Dec 2021.

We used a convergent interviewing technique [48], conducting interviews in person (pre-pandemic) or via telephone or a virtual platform (Cisco Webex, Zoom or Microsoft Teams), depending on the preference of the participant. Convergent in-depth interviews are characterized by a structured process and unstructured content. Interviews are embedded within a process of design and analysis so that subsequent interviews can build on reflective opportunities from former interviews. Interview questions were designed to investigate: the participant’s definition of patient trust and the uniqueness of the doctor–patient relationship; their trust in other individuals and institutions, and how this is similar to or differs from healthcare providers and the healthcare system; their current trust in the Canadian health care system, whether they trust without reservation, and if so, why; previous experiences with the system, and how this influences their perception of the system; how their social identity (related to equity-deserving populations) affects their trust; and how their trust might be (re)built/maintained/restored. All participants were also asked to complete and comment on V1 of the measure for content validity and speak to difficulties in understanding draft items.

Interviews were recorded and transcribed verbatim by an agency abiding by a confidentiality agreement. We then underwent a process of conceptual coding with the goal of revising candidate items for version 2 (V2). In brief, analysts inductively and deductively coded data to identify both existing and emerging dimensions of trust. Conceptual categories were grouped to align with dimensions included in V1 (see Table 1) and items were reviewed, edited, and removed. New items were created based on inductive codes. Any discrepancies between the data and V1 of the measure were documented, leading to V2 for use in the expert validation process.

**Table 1:** Existing dimensions

Measure	Dimensions
Wake Forest Physician Trust Scale [37]	Fidelity Competence Honesty Confidentiality Global trust
Public Trust in Dutch Healthcare [40]	Patient focus of providers Policies at the macro level will be without consequences for the patient Health care providers’ expertise Quality of care Information supply and communication by care providers Quality of cooperation

#### Phase 3 methods: expert validation of items

A virtual meeting (due to COVID-19 travel restrictions) was held with international co-investigators (Calnan, Ward, and Brown) with expertise in the sociology of trust for the purpose of establishing face validity. In advance of the meeting, the lead investigator prepared a summary document containing: (1) Findings from qualitative interviews demonstrating how V1 dimensions and related items mapped to the qualitative data; (2) The original (V1) and revised measure (V2), including documented rationale for the creation/removal or modification of items; (3) An overview of the planned process for the construct validation process (e.g., items for convergent or discriminant validity); and (4) A list of outstanding items for consideration based on the conceptual expertise of the team (e.g., missing constructs to be used for discriminant validity, ideas for criterion validity). An exercise related to this summary document was sent to international experts to complete through Google Forms in advance of the meeting to facilitate documentation processes.

The validation of items occurred during the three-hour meeting, held October 28th, 2021. Experts systematically reviewed individual responses to the exercise, whereby all participants presented their response and rationale for discussion. The lead author documented each decision made by the team (e.g., the finalised list of constructs for discriminant validity), to keep record of the conceptual or theoretical rationale behind these decisions. The lead author finalized the survey for circulation and approval in advance of the validation study.

### Results of development study

#### Phase 1 results: systematic review (item generation)

Through our review, two measures were selected as the starting point for our work validating a multidimensional model of trust; the Wake Forest Physician Trust Scale [37] and the Public Trust in Dutch Healthcare measure [40]. These measures were chosen for their conceptual foundations (e.g., building on foundational scales of trust including [42, 61]) and inclusion of items that have been used inother contexts within healthcare, including oncology [49], nursing, emergency departments [50], pediatrics, health insurance [51], rheumatology [52], medical researchers [53], and clinical research [54]. Additionally, these measures have been adapted to look at levels of trust in specific population groups including parents [55], racial minorities [54, 56], and the elderly [57]. Both have demonstrated strong reliability and validity, with a Cronbach’s alpha of 0.9 [37] and 0.8 [40], respectively. Scores from the Public Trust in Dutch Healthcare measure have been found to correlate with patient experience, quality of care, and patient-centred care [40, 58]. Scores on the Wake Forest Physician Trust Scale have been found to correlate strongly with satisfaction, desire to remain with a physician, willingness to recommend to friends, and not seeking second opinions [37, 59]. These measures were developed over twenty years ago and as we note above, fall into the list of measures with notable conceptual and methodological limitations. As such, we used the dimensions (see Table 1) and related survey items from each of these measures as solely a starting point for the development of V1 of our measure. We extend these respected measures for use in present-day with diverse populations, accounting for noted conceptual and methodological limitations.

#### Phase 2 results: qualitative interview (modification of items)

Our analysis of qualitative data confirmed all existing dimensions of trust. However, some questions from the Straten et al. measure were noted to be unclear in terms of wording, which may be because the instrument was originally developed for use in the Netherlands where the meaning of trust in Dutch was translated as reflecting confidence. As such, we adapted some of wording used by Calnan and Sanford [58], a team that drew on but adapted the Public Trust in Dutch Healthcare to measure institutional trust in the National Health Service, England. For example, the item ‘Cost-cutting will not be at the disadvantage of patients’ was reworded to state ‘Cost cutting does not disadvantage patients’. This language was determined by the team to better reflect Canadian terminology. We also removed the lead-in statement used by Straten (“I have absolute confidence that” followed by statements upon which a respondent agrees/disagrees). Rather, we began each statement with “I trust that…” and instead added a lead-in to each of Straten’s original six dimensions, noting to respondents, for example, that “The following questions are related to your perception regarding the information you receive about your health and communication with healthcare providers. Please indicate the extent to which you agree with the following statements.”

In reviewing interview data, we also identified specific indicators of trust that were critical to participants’ trust. For example, in response to data regarding trust as it relates to not being judged by doctors (e.g., a participant stated “Your provider can want the best for you while not supporting your lifestyle”) we added the item ‘I trust that doctors do not judge their patients’. In this sense, while dimensions remained consistent, specific items relevant to Canadians’ trust were developed and incorporated into existing measures. In total, 16 items were added to the measure, all falling under existing dimensions (item generation and deletion, and rationale, available upon request). To reflect interviewee statements about the wording of questions, we also changed the scaling of items, moving to a Likert scale looking at level of agreement, rather than a scale towards complete trust (e.g., it was noted that ‘completely’ trust to ‘somewhat’ trust is not granular enough). Version 2 (V2) was then used in the expert validation process. 

#### Phase 3 results: Expert validation of items

During the expert review process, the team came to a consensus on the modification of items in V2 on the basis of qualitative data (e.g., it was proposed that we consider adding an item related to reliability and dependability, leading to additional items under the dimension of patient focus of providers) and generated a final list of items for the validation process that, in some cases, involved developing new questions (e.g., for criterion validity we used existing measures of satisfaction but developed items related to dependence). It was also determined that the dimensions proposed by Hall et al [37] were too highly correlated with the Straten et al. [40] dimension of patient focus of providers. The 11-item scale was thus removed from the analysis and instead included for the purpose of convergent validity (discussed below). Detailed notes were recorded, and all changes were reflected in version 3 (V3) for use in the validation study.

### Conclusion of the Development Study

Based on a 2021 review of existing measures [11], 72 qualitative interviews, and an expert review consensus process, 43 items were considered for the validation process described below, covering the six dimensions: Patient focus of providers; Policies at the macro level; Health care providers’ expertise; Quality of care; Information supply and communication by care providers; Quality of cooperation. Table 2 lists the 43 items. The number of questions as they appear will be referenced in our analyses.

**Table 2:** Version 3 measure items

Dimension	Items (*N* = 43)
[Q12] Patient focus of providers (5-point Likert scale)	[a] I trust that doctors put patients’ interests ahead of their own [b] I trust that doctors treat all patients the same [c] I trust that doctors do not judge their patients [d] I trust that doctors are responsive to feedback they receive from their patients [e] I trust that doctors do not take advantage of their patients [f] I trust that patients are taken seriously [g] I trust patients get enough attention [h] I trust that patients are listened to [i] I trust that doctors spend enough time on their patients [j] I trust that doctors will always stick up for their patients [k] I trust that doctors can relate to their patients’ problems [l] I trust that doctors will be consistent in the care they provide [m] I trust that doctors trust me
[Q13] Policies at the macro level will be without consequences for the patient	[a] I trust that the health system has the staffing and resources needed to provide the care Canadians need [b] I trust that the privatization of health care services does not disadvantage patients [c] I trust that healthcare will be affordable for me [d] I trust that medical help and patient care will not be compromised by waiting lists [e] I trust that patients will not be the victims of the rising costs of health care [f] I trust that waiting times are never too long [g] I trust that doctors have control over the decisions they make about my care
[Q14] Health care providers’ expertise	[a] I trust that doctors will admit when they have made mistakes [b] I trust that doctors are committed to continuing their education and training [c] I trust that doctors are knowledgeable about a range of diseases [d] I trust that new treatments are put into practice in the healthcare system [e] I trust that the education and training of doctors in this country is one of the world’s best [f] I trust that doctors will continue to respond to new and emerging medical problems
[Q15] Quality of care	[a] I trust that patients always get the right dose of medicine [b] I trust that patients are referred to specialists in time [c] I trust that patients always get the right type of medicine [d] I trust that doctors will prescribe medicines at the appropriate time (not too early or too late) [e] I trust that patients’ medical information is kept confidential [f] I trust that doctors do enough tests (not too few or too many) [g] I trust that patients will always get the best treatment [h] I trust that doctors will make the right diagnosis
[Q16] Information supply and communication by care providers	[a] I trust that the information given to patients is clear and understandable [b] I trust that patients get sufficient information about the cause of their problems [c] I trust that doctors discuss things fully with their patients [d] I trust that patients get sufficient information about the various treatment options that are available [e] I trust that patients get sufficient information about the effects of their treatment [f] I trust that doctors make use of the patients’ own understanding and insights
[Q17] Quality of cooperation	[a] I trust that healthcare providers are good at cooperating with each other [b] I trust that patients are not given conflicting information [c] I trust that high levels of specialisation benefits the healthcare system

## Validation of the trust in multidimensional healthcare systems scale (TIMHSS)

### Methods for validation study

#### Approach to convergent, discriminant and criterion validity

In addition to the items measuring trust, V3 included demographic questions (Q1-11 and 29: province, gender and sexual orientation, age, social identity [e.g., membership with community groups], country of birth, years living in Canada, ethnicity, income, education, political affiliation, religion, health status) to identify if the factor structure of our measure was consistent across demographic groups, particularly given that, as we discuss below, we oversampled equity-deserving groups who we hypothesised to be least trusting (individuals who identify as women or non-binary, a racial minority, and do not identify as heterosexual).

An additional nine questions were included for the purpose of testing convergent, discriminant, and criterion validity (see Table 3). For criterion validity, we analysed the extent to which trust predicts anticipated behaviours identified in the literature; namely, acceptance of medications or treatment plans [40, 41, 60, 61], delaying access to care, disclosure of medically relevant information [10, 62] and not requesting a second opinion [40]. Considering the COVID-19 pandemic, we added uptake of new vaccines. For convergent validity, we identified existing items/scale(s) that *should* correlate with our scale to demonstrate validity of the instrument. Satisfaction served this purpose as we would expect these constructs to correlate. We also included the Wake Forest Physician Trust Scale [Q18] [37], expecting our measure, and particularly items related to providers and the system, to be highly correlated. For discriminant validity, we measured dependence based on research demonstrating the semantic distinctions between these two distinct concepts [26–28]. As there was no available existing measure of dependence, two items were developed for use and ratified with team members.

**Table 3:** Validation items

Question	Contribution to validation (citation of measure, where applicable)
[Q19] I never question the medical advice I am given by my doctor. (agree/disagree)	Discriminant validity – Dependence
[Q20] I have no choice but to follow the recommendations provided by my doctor. (agree/disagree)	Discriminant validity – Dependence
[Q21] I always follow doctors’ recommendations. (agree/disagree)	Criterion validity - Acceptance of medication or treatment plan
[Q22] I would be willing to accept a new vaccine if my doctor recommended it. (agree/disagree)	Criterion validity - Uptake of new vaccines
[Q23] During the past 12 months, was there any time when you didn’t get the medical care you needed (yes/no)	Criterion validity – Delay in access to care
[Q24] I always tell my doctor the truth when they ask for information relevant to my healthcare. (yes/no)	Criterion validity – Disclosure of medically relevant information [63]
[Q25] Have you changed physicians in the past or sought a second opinion due to concerns about care? (yes/no)	Criterion validity – Request for second opinion [37]
[Q26] I am perfectly satisfied with the health care I have been receiving. (strongly agree to strongly disagree)	Convergent validity – Satisfaction [64]
[Q27] There are some things about the health care I have been receiving that could be better. (strongly agree to strongly disagree)	Convergent validity – Satisfaction

#### Study sample

Two waves of participants living in Canada ages 18 + were recruited through Leger for the purpose of measure validation. Wave 1 (exploratory) data were collected between November 15th – 29th 2021 (*N* = 694) and analysed using exploratory factor analysis. Wave 2 (validation and confirmatory) data were collected between January 27th – February 4th, 2022 (*N* = 740) and used to confirm the factor structure, consistency in structure across population subgroups, and its association with other measures. Leger over sampled in both waves for identified equity-deserving populations using stratified random sampling. Sample size calculation was done based on a regression-style analysis with power of 0.8, significance value of 0.05 and 5 explanatory variables, which would require a minimum of ∼ 400 participants.

#### Statistical analysis

For both the exploratory and validation data sets, descriptive statistics for each item were first examined by calculating the mean, standard deviation (SD), skewness, and kurtosis values. If most items were not highly skewed, the Pearson’s correlation matrix was specified for further analysis.

For exploratory factor analysis (EFA), sampling adequacy was established first using the Kaiser-Meyer-Olkin (KMO) test, Bartlett’s Test of Sphericity, and the Shapiro-Wilk test of normality. If these statistics indicated that the dataset was suitable for EFA, the eigenvalues and scree plot were retrieved next to determine the number of factors that should be tested. If more than one factor was present, orthogonal and oblique rotation options were considered. After deciding on the number of factors and rotation methods to test, the corresponding EFA models were constructed. Factor loadings, average inter-item correlations (IIC), Cronbach’s alpha, and communality/uniqueness values were examined for each model. Items that performed poorly (factor loading < 0.30, cross-loading > 0.30, high uniqueness values) were removed from analysis, according to the following criteria: primary factor loading less than 0.30, cross-loadings greater than 0.35, and uniqueness values greater than 0.60 [65].

Based on the results from the EFA, numerous models with varying numbers of factors were created for confirmatory factor analysis (CFA) and Structural Equation Modeling (SEM). To determine model performance, fit statistics were calculated for each model. Established scoring conventions were used to set minimum requirements for fit statistics, including values of > 0.90 on the Comparative Fit Index (CFI), < 0.06 on the Root Mean Square Error of Approximation (RMSEA), and < 0.08 on the Standardized Root Mean Squared Residual (SRMR) [66]. To compare the fit across models, the Akaike information criterion (AIC) and Bayesian information criterion (BIC) were consulted. Combined with theoretical relevance, fit statistics were used to select a preferred model.

#### Validity tests

To perform validity and measurement invariance tests, it was necessary to retrieve the estimated factor scores for each individual, given the structure of the model chosen. To obtain factor scores, the lavPredict function within the Lavaaan package for R version 4.1.2 was used, with the default scoring method specified (regression coefficient).

*Convergent validity.* Two questions measuring satisfaction (see Table 3) were included in the survey to determine convergent validity: Q26 (‘I am perfectly satisfied with the health care I have been receiving’) and Q27 (‘There are some things about the health care I have been receiving that could be better’). As noted, the Wake Forest Physician Trust Scale [37] was also included for the purpose of convergent validity. To establish the association between validity items and our three-factor TIMHSS, Spearman rank correlation analyses were performed.

*Discriminant validity.* Two questions (see Table 3) were included in the survey to determine discriminant validity from dependence: Q19 (‘I never question the medical advice I am given by my doctor’) and Q20 (‘I have no choice but to follow the recommendations provided by my doctor’). Point biserial correlations were calculated between each of the three factors in the TIMHSS and Q19 and Q20.

*Criterion validity.* For each of the five criterion validity questions (see Table 3, Q21-Q25) administered with the survey, logistic regression models were performed using global health trust survey scores as the sole predictor (Table 4). To create a global score, factor scores were summed across each individual.

**Table 4:** Binary logistic regression models with global health care trust scores as the sole predictor

Outcome variable	Reference group	Slope estimate	Standard error	z-value	p-value
Always follow doctors’ recommendations	Disagree	0.49	0.05	9.15	< 0.0001
Would be willing to accept a new vaccine if my doctor recommended it	Disagree	0.41	0.06	7.30	< 0.0001
In last 12 months, chose not to get needed medical care	No	− 0.33	0.05	-5.98	< 0.0001
Always tell the doctor the truth about health information	No	0.21	0.09	2.47	0.01
Previously changed physicians or asked for second opinion due to concerns about care	No	− 0.30	0.05	-5.99	< 0.0001

*Note* Global health care trust scores were calculated using factor scores derived from the three-factor SEM.

#### Measurement of invariance

To determine whether the model was consistent across demographic groups, measurement invariance was tested for women vs. non-women, as well as diverse gender and sexual orientation groups vs. cisgender and heterosexual group. These demographic groups were selected because they had adequate sample sizes for comparing complex models. Based on previously established conventions for establishing measurement invariance [67], configural, metric, and scalar invariance models (full and partial) were compared for both groups.

After establishing measurement invariance, the distribution of each survey question was also compared for a combined equity-deserving group, including individuals identifying themselves as: women, of diverse gender identities and sexual orientations, ethnicities other than White, and/or member of a visible minority. All data analyses were performed using R version 4.1.2 [68]. The list of R packages used in the analysis include: psych [69], lavaan [70], mvnormtest [71], EFAtools [72], semTools [73], and performance [74].

Ethics approvals were granted by the University of Waterloo Research Ethics Borad. Ethical considerations of informed consent/anonymity/confidentiality/data security were observed for all phases of the project.

### Results of validation study

#### Sociodemographic characteristics

The sociodemographic characteristics of both Wave 1 and 2 samples are provided below in Table 5. Overall, the patterns of characteristics are similar between the two samples.

**Table 5:** Sociodemographic characteristics of Wave 1 (*N* = 694) and Wave 2 (*N* = 720) samples

Variable	Response option	Wave 1 (*N* = 694) % of sample (n)	Wave 2 (*N* = 740) % of sample (n)
Gender identity	Man	42.5 (299)	45.7 (338)
	Woman	56.0 (389)	52.7 (390)
	Agender	0.1 (1)	0.1 (1)
	Bigender	0.3 (2)	0.3 (2)
	Genderqueer / Gender non-conforming / Gender non-binary	1.0 (7)	0.9 (7)
	Intersex	0.0 (0)	0.0 (0)
	Pangender	0.0 (0)	0.1 (1)
	Trans	0.4 (3)	0.7 (5)
	Two-spirit	0.6 (4)	0.1 (1)
	Queer	0.4 (3)	1.8 (13)
	Another gender identity not listed	0.1 (1)	0.3 (2)
Sexual orientation	Heterosexual	78.2 (543)	76.5 (566)
	Gay man	6.0 (42)	4.2 (31)
	Lesbian	1.7 (12)	2.0 (15)
	Bisexual / Pansexual	9.2 (64)	11.1 (82)
	Asexual / Aromantic	1.4 (10)	2.2 (16)
	Questioning	1.0 (7)	1.3 (10)
	Another sexual orientation not listed	1.1 (8)	1.6 (12)
Ethnicity	Caucasian	55.6 (386)	68.6 (508)
	Asian	10.9 (76)	9.2 (68)
	First Nation, Inuit, Metis	13.3 (92)	8.4 (62)
	Black / African Canadian	17.1 (119)	9.7 (72)
	South / Central American	0.6 (4)	0.3 (2)
	Arab	0.4 (3)	0.8 (6)
	Another ethnicity not listed	2.0 (14)	3.0 (22)
Age group	18–24	14.3 (99)	14.7 (109)
	25–34	23.5 (163)	18.1 (134)
	35–44	18.6 (129)	15.7 (116)
	45–54	21.8 (151)	15.8 (117)
	55–64	12.1 (84)	15.4 (114)
	65–74	7.2 (50)	15.5 (115)
	75–84	2.3 (16)	4.6 (34)
	85+	0.2 (2)	0.1 (1)
Gross household income	<$19,999	6.8 (47)	13.2 (98)
	$20,000 - $39,999	16.1 (112)	17.3 (128)
	$40,000 - $59,999	17.3 (120)	13.6 (101)
	$60,000 - $79,999	15.0 (104)	10.8 (80)
	$80,000 - $99,999	12.4 (86)	13.0 (96)
	$100,000 - $119,999	8.5 (59)	9.6 (71)
	$120,000 - $139,999	4.2 (29)	5.0 (37)
	$140,000 - $159,999	3.6 (25)	4.3 (32)
	>$160,000	8.8 (61)	5.9 (44)
	Prefer not to answer	7.3 (51)	7.2 (53)

*Note* Multiple options could be selected for gender identity, and so percentages may exceed 100

#### Item descriptive statistics

Item means in the Wave 1 dataset ranged from 2.11 to 3.74, with standard deviations (SD) ranging from 0.89 to 1.27. Average scores were similar in the Wave 2 dataset: item means ranged from 2.05 to 3.83, with SDs of 0.93 to 1.31. While median values ranged from 2 to 4 in both datasets, most items had a median score of 2, demonstrating that questions generally scored towards the middle-low end of the distribution. Item skewness ranged from − 0.69 to 1.04 (*M* = 0.45) in the wave one dataset and − 0.84 to 1.00 (*M* = 0.42) in wave two, with only two items demonstrating a skewness value of 1 or greater (17b and 17c). In waves one and two, kurtosis values ranged from − 1.17 to 1.46 (*M=*-0.31) and − 1.09 to 1.08 (*M=*-0.32), respectively, suggesting that distribution of scores are somewhat symmetrical. Overall, survey responses were slightly skewed towards the lower-middle range in both datasets.

Pearson’s correlation coefficients ranged from − 0.41 to 0.79, with an average inter-item correlation (IIC) of 0.50, suggesting that survey questions are moderately related to one another. The Cronbach’s alpha for the correlation matrix was 0.98, though this estimate may be inflated due to the large number of items present in the survey [75]. The correlation matrix is provided in Additional file 1.

#### Sampling adequacy

After excluding cases that contained missing values for any of the survey items, the total sample size was *n* = 622, exceeding the minimum guideline of 300 [76]. Furthermore, the MSA value for the correlation matrix was 0.98, which is considered high [77]. Similarly, Bartlett’s Test of Sphericity [78] was significant at 𝜒²(903) = 22,457.53, *p* <.001, indicating that the dataset was appropriate for EFA.

**Fig. 1:**
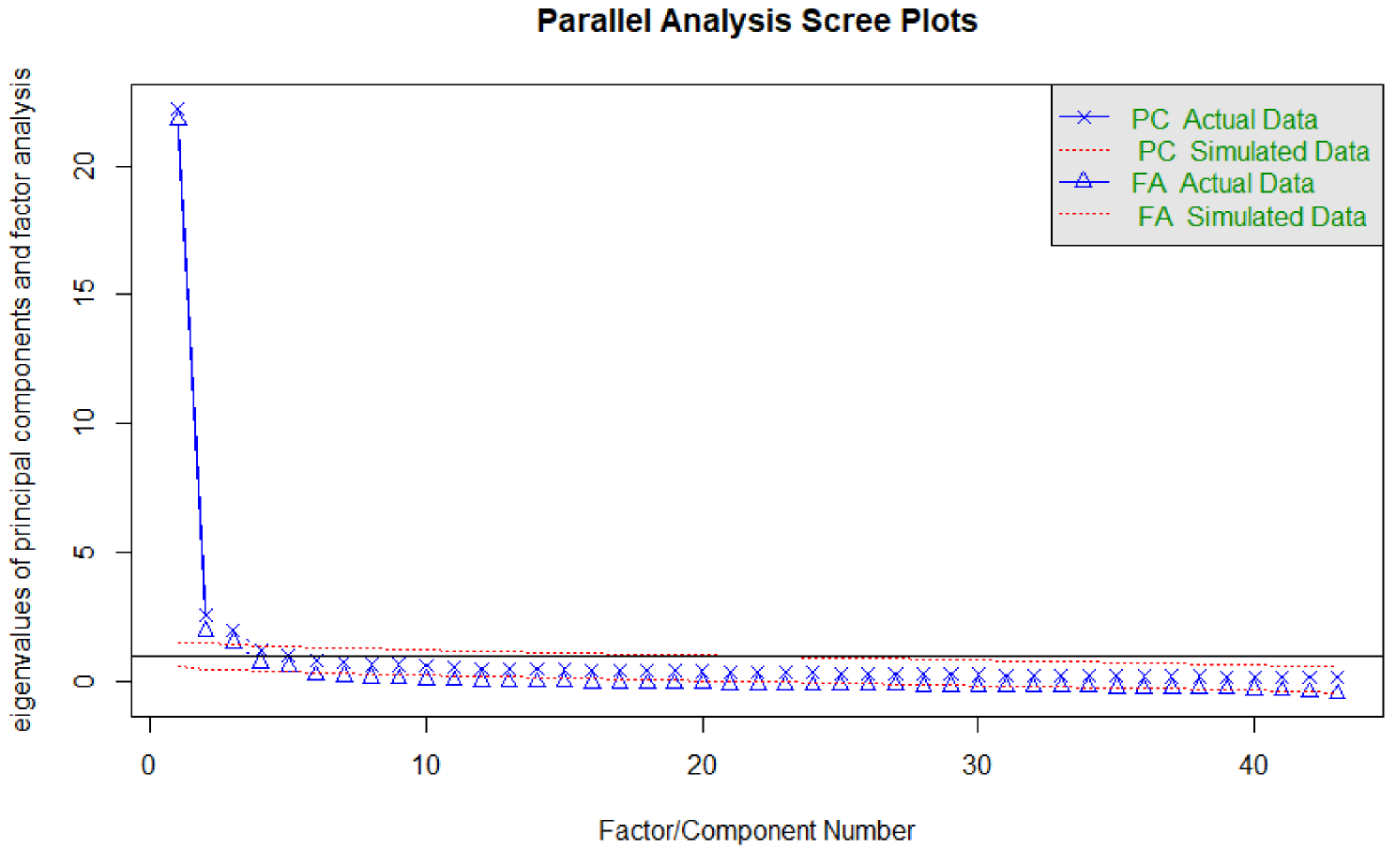
Parallel analysis eigenvalues and scree plots for 43 health care trust survey questions

The parallel analysis scree plot (Fig. 1) suggests that there is one major factor accounting for a large portion of variance, with up to three additional factors. Eigenvalues for the top five factors were, respectively: 22.24, 2.59, 1.98, 1.21, and 1.03. Based on the eigenvalues and parallel analysis scree plot, it was decided that up to four factors would be extracted for the EFA models.

#### EFA models

The distribution of the dataset was multivariate non-normal (*W* = 0.82, *p* <.0001), and so the principal factors (PA) estimation method was used for extracting factors for the exploratory models [79].

Based on the scree plots and eigenvalues, several models were created with varying numbers of factors, as well as rotation methods. In a unidimensional model with all 43 survey items (see Table 2), aside from Q13b, all items returned factor loadings above 0.40, suggesting that a unidimensional factor merits exploration in the CFA. Results for the unrotated two-, three- and four-factor models, as well as orthogonal two- and three-factor models, are available upon request.

In addition to unrotated and orthogonal rotations, a series of models with oblique rotations were also conducted. In the two-factor model, all items from Q13 formed a separate factor (Factor 2), except for questions 13c and 13 g, the latter of which loaded onto factor one. Question 13c did not load onto either factor and had the second highest uniqueness value (0.62), indicating that it is not well-related to the latent factor structure. Similarly, despite loading onto Factor 2 (0.52) 13b was removed due to having the highest uniqueness score (0.70). Like the two-factor model, Q13b and Q13c demonstrated high uniqueness values, with Q13c failing to load strongly onto any factor. In the three-factor model, Q13g, Q14a and Q15b also failed to load onto one factor. To determine whether these four questions adversely affected the internal consistency of the model, changes to the Cronbach’s alpha and IIC were compared before and after each one was removed. The Cronbach’s alpha remained unchanged after removing each item, though the IIC was slightly lower when retaining Q13b and Q13c. Due to poor factor loadings and high uniqueness values, these five questions were removed from the three-factor model, leaving 38 questions altogether. The final rotated three-factor solution is presented in Table 6.

**Table 6:** Three-factor EFA model with oblique rotation

Survey question	System	Policy	Doctor	Uniqueness
Q12a-I trust that doctors put patients’ interests ahead of their own			0.59	0.50
Q12b-I trust that doctors treat all patients the same			0.89	0.33
Q12c-I trust that doctors do not judge their patients			0.78	0.36
Q12d-I trust that doctors are responsive to feedback they receive from their patients			0.81	0.35
Q12e-I trust that doctors do not take advantage of their patients			0.65	0.43
Q12f-I trust that patients are taken seriously			0.79	0.28
Q12g-I trust patients get enough attention			0.82	0.28
Q12h-I trust that patients are listened to			0.83	0.26
Q12i-I trust that doctors spend enough time on their patients			0.78	0.34
Q12j-I trust that doctors will always stick up for their patients			0.77	0.27
Q12k-I trust that doctors can relate to their patients’ problems			0.69	0.40
Q12l-I trust that doctors will be consistent in the care they provide			0.73	0.28
Q12m-I trust that doctors trust me			0.70	0.34
Q13a-I trust that the health system has the staffing and resources needed to provide the care Canadians need		0.62		0.50
Q13d-I trust that medical help and patient care will not be compromised by waiting lists		0.83		0.24
Q13e-I trust that patients will not be the victims of the rising costs of health care		0.70		0.37
Q13f-I trust that waiting times are never too long		0.78		0.33
Q14b-I trust that doctors are committed to continuing their education and training	0.64			0.49
Q14c-I trust that doctors are knowledgeable about a range of diseases	0.77			0.44
Q14d-I trust that new treatments are put into practice in the healthcare system	0.70			0.46
Q14e-I trust that the education and training of doctors in this country is one of the world’s best	0.83			0.46
Q14f-I trust that doctors will continue to respond to new and emerging medical problems	0.84			0.40
Q15a-I trust that patients always get the right dose of medicine	0.61			0.41
Q15c-I trust that patients always get the right type of medicine	0.65			0.37
Q15d-I trust that doctors will prescribe medicines at the appropriate time (not too early or too late)	0.61			0.37
Q15e-I trust that patients’ medical information is kept confidential	0.77			0.49
Q15f-I trust that doctors do enough tests (not too few or too many)	0.56			0.41
Q15g-I trust that patients will always get the best treatment	0.56			0.29
Q15h-I trust that doctors will make the right diagnosis	0.63			0.34
Q16a-I trust that the information given to patients is clear and understandable	0.67			0.41
Q16b-I trust that patients get sufficient information about the cause of their problems	0.59			0.40
Q16c-I trust that doctors discuss things fully with their patients	0.51			0.37
Q16d-I trust that patients get sufficient information about the various treatment options that are available	0.62			0.35
Q16e-I trust that patients get sufficient information about the effects of their treatment	0.70			0.36
Q16f-I trust that doctors make use of the patients’ own understanding and insights	0.55			0.36
Q17a-I trust that healthcare providers are good at cooperating with each other	0.58			0.54
Q17b-I trust that patients are not given conflicting information	0.53			0.46
Q17c-I trust that high levels of specialisation benefits the healthcare system	0.75			0.54

*Note* Oblimin rotation specified. Items were standardized prior to running the factor analysis

Since all four items that loaded onto Factor 3 were from question 13, this factor was named the ‘Policy’ factor. Factor 2 was comprised entirely of the 13 items from Q12, with all factor loadings above 0.40 and no cross-loading onto any other factor. Since Q12 was focused on trust in service doctors specifically, this factor was called the ‘Doctor’ factor. Finally, the remaining 21 items belonged to Factor 1. Unlike the Doctor and Policy factors, Factor 1 addressed a variety of systemic health care issues, and so this was named the ‘System’ factor.

The Doctor and System Factors were highly correlated (*r* =.78), whereas the Policy factor was moderately correlated with both Doctor (*r* =.42) and System (*r* =.50). The Doctor and System factors explained more of the variance (0.29 and 0.24, respectively), though all three factors combined explained 0.62 of the total item variance. The raw Cronbach’s alpha values for each of the three factors were: Doctor (α = 0.96), Policy (α = 0.88), and System (α = 0.96).

#### CFA models

Based on the 38 items that were retained after conducting EFA, the following models were constructed for CFA: unidimensional factor, correlated two and three-factors, and hierarchical two- and three-factor models (results available upon request). Given that the assumption of multivariate normality was violated, the estimation method used for deriving models was maximum likelihood (ML) estimation with robust standard errors and a Satorra-Bentler scaled test statistic, which performs better than regular ML when data is nonnormal [80]. Although each model was over-identified and factor loadings for items exceeded 0.40, fit indices did not meet the recommended scoring criteria in any model. Upon examining the standardized residual errors between the observed and expected covariance matrices, it was apparent that there was unexplained variance remaining between items. To establish the sources of model misspecification, the top 10 parameters contributing to poor model fit were computed using the ‘modindices’ function in the Lavaan package for R. These tests suggested that model fit would improve if various item error variances were permitted to correlate. To accommodate correlated residual error terms, analysis pivoted to Structural Equation Modeling (SEM).

#### Structural equation modelling

Model fit indices derived from CFA structures suggested that residual error terms for various items be allowed to correlate, particularly within question blocks 12 (two-factor model only) and 14. To account for shared error terms, one-, two-, and three-factor SEMs were constructed, with covarying residual terms specified for items within question blocks. In each of the models, covarying error terms that were insignificant at the *p* <.0001 level and had a standardized correlation value lower than 0.20 were removed from the model in a backwards elimination approach. Altogether, in the three-factor model, 36 covarying residual error terms were required to establish acceptable model fit. For two-factors, an additional 60 shared error terms were needed (all items in question 12), totaling to 96. Finally, the unidimensional model required covarying residual terms for items in Q13, resulting in 100 shared error terms. Given the undesirability of correlated error terms in SEM arising from increased model complexity and difficulty in replicating parameters across populations [81], and that the pattern of correlated error terms in the one- and two-factor models matched item-groupings in the three-factor model, the three-model factor was selected as the preferred structure. Model fit indices for the three-factor model (DF = 755) were acceptable (CFI = 0.93, RMSEA = 0.05, SRMR = 0.05). The factor loading table for the final three-factor model is provided in Additional file 2.

#### Validity tests

*Convergent validity.* The correlation coefficients for Q26 were *r*_*s*_=0.57 (*p* <.0001) for the Doctor factor, *r*_*s*_=0.61 (*p* <.0001) for the System factor, and *r*_*s*_=0.35 (*p* <.0001) for the Policy factor, demonstrating a moderate association between the Doctor and System factors and satisfaction with care received. The correlation coefficients for Q27 were weaker: *r*_*s*_=0.30 (*p* <.0001) for the Doctor factor, *r*_*s*_=0.33 (*p* <.0001) for the System factor, and *r*_*s*_=0.28 (*p* <.0001) for the Policy factor. Finally, the correlation coefficients between the Wake Forest Physician Trust Scale [37] and the Doctor, System, and Policy factors were, respectively: *r*_*s*_=0.52 (*p* <.0001), *r*_*s*_=0.53 (*p* <.0001), and *r*_*s*_=0.14 (*p* <.0001). As expected, these results suggest that there is convergent validity between the Doctor and System factors within the TIMHSS and satisfaction with health care provision, and also the Wake Forest Physician Trust Scale [37], but not the Policy factor.

*Discriminant validity.* For Q19, the correlation coefficients were as follows for the Doctor, System, and Policy factors, respectively: *r*=-.34 (*p* <.0001), *r*=-.38 (*p* <.0001), and *r*=-.28 (*p* <.0001). The correlation coefficients between the Doctor, System, and Policy factors and Q20 were as follows: *r*=-.10 (*p* <.01), *r*=-.14 (*p* =.0001), and *r*=-.15 (*p* <.0001). The results of these analyses suggest that there is weak association between the TIMHSS and not questioning medical advice, and a negligible relationship with feeling as though there is no choice but to follow a doctor’s recommendation, supporting the hypothesis that our measure is distinct from measures of health care dependence.

*Criterion validity*. Global factor scores on the TIMHSS significantly predicted each of the criterion validity questions included in the study. Further, the direction of effect for all outcome variables were consistent with a priori hypotheses. For instance, higher trust scores were related to greater log odds of telling doctors the truth about health information, but lower log odds of not seeking medical care when needed. The results of these regression models suggest that the TIMHSS is consistently predictive of theoretically relevant outcomes, demonstrating support for criterion validity.

#### Item distributions for equity-deserving populations

To ensure that the survey is an accurate representation of trust in the health system for equity-seeking populations, the internal consistency of the factor structure was tested across multiple demographic groups. The first step was to compare item distributions between equity-deserving and reference groups using chi-square tests (available upon request). Notably, the only question in which many items differed significantly between the two groups was Q12 (Doctor factor), further supporting the idea that a three-factor model is more useful than a two-factor model.

#### Measurement invariance across demographic subgroups

*Non-women.* Of the total sample (*N* = 740), 390 individuals identified as women and 350 did not. Given that the sample size was similar for both groups, analysis proceeded with the full sample.

In the configural models for women and non-women, all factor loadings were above 0.50. Further, the difference between factor loadings was minimal between the two groups, ranging from 0.0 to 0.10.

As observed in Table 7, according to the probability value of the chi-square test, there was insufficient support for full metric and scalar invariance of the three-factor model. As a result, partial metric and scalar invariance models were created. Slopes and intercepts contributing to model invariance were identified using the partial Invariance test in the semTools package of R.

**Table 7:** Comparison of original, metric, scalar, and residual invariant SEM three-factor models for women (*n* = 390) vs. non-women (*n* = 350)

Model	DF	Chi-square	RMSEA	CFI	SRMR	AIC
Three-factor	1,288	3,294.3	0.055	0.937	0.047	62,265.19
Full metric invariance	1,323	3,361.9***	0.055	0.935	0.057	62,262.72
Full scalar invariance	1,358	3,436.4***	0.055	0.933	0.058	62,267.24
Full residual invariance	1,396	3,506.0	0.054	0.932	0.058	62,260.85
Partial metric invariance	1,318	3,336.9	0.054	0.936	0.053	62,247.71
Partial scalar invariance	1,344	3,366.7	0.054	0.936	0.054	62,225.55

*Note* Nested model comparisons using scaled chi-squared difference test with Satorra-Bentler estimation method. Robust test statistics reported for RMSEA, CFI, and SRMR.

**p-value of chi-square difference test between original and full metric model is < 0.001

***p-value of chi-square difference test between full metric and full scalar model is < 0.0001

For the partially invariant metric model, variables that substantially reduced the chi-square statistic when factor loadings were set to be freely estimated were: Q12e, Q11f, Q15a, Q15f, Q16c, and Q16e. Altogether, this represents 17% of items in the survey (7/38). Regarding partial scalar invariance, the following intercepts led to substantial reductions in the chi-square statistic when freely estimated: Q12a, Q14b, Q14d, Q15d, Q15e, Q15f, Q16a, Q17a, and Q17c. In this case, 24% of items in the survey required free intercepts (9/38). The chi-square difference test between the partial metric and partial invariance models was statistically insignificant (p ≥.05). Further, model fit statistics did not differ substantially between the models according to the guidelines previously described, indicating that, with some adjustments, the survey is a valid representation of trust in the health care system for women and non-women.

To determine the effect of identifying as a woman on global health care trust scores, factor scores of the partially invariant scalar model were retrieved for each individual. In a linear regression model, identifying as a woman was associated with a significant decrease in health care trust (β=-0.48, SE = 0.15, *t*=-3.30, *p* =.001).

*Diverse gender and sexual orientation group vs. Cisgender and heterosexual group.* The sample size of individuals in the diverse gender and sexual orientation group was *n* = 166. To create an equal sample size, a random selection of *n* = 166 individuals in the cisgender and heterosexual group was selected using the ‘sample’ command in R using a set seed of 23.

Comparison between the configural models (see Table 8) for the diverse gender and sexual orientation group and cisgender and heterosexual group revealed that all factor loadings remained above 0.50, and that differences in factor loadings were minimal (ranging from 0.0 to 0.1).

**Table 8:** Comparison of original, metric, scalar, and residual invariant SEM three-factor models for diverse gender and sexual orientation group (*n* = 166) vs. random sample of cisgender and heterosexual group (*n* = 166)

Model	DF	Chi-square	RMSEA	CFI	SRMR	AIC
Three-factor	1,288	2,722.6	0.065	0.913	0.058	28,449.73
Full metric invariance	1,323	2,751.3	0.064	0.913	0.065	28,408.43
Full scalar invariance	1,358	2,789.9*	0.064	0.912	0.066	28,377.06
Full residual invariance	1,396	2,877.6*	0.064	0.910	0.068	28,388.67
Partial scalar invariance	1,355	2,782.6	0.063	0.913	0.066	28,375.66

*Note* Nested model comparisons using scaled chi-squared difference test with Satorra-Bentler estimation method. Robust test statistics reported for RMSEA, CFI, and SRMR.

*p-value of chi-square difference test between full metric and full scalar model is < 0.001

*p-value of chi-square difference test between full scalar and full residual model is < 0.05

The chi-square difference test between the original and full metric invariance models was not statistically significant (*p* >.05), suggesting that survey items loaded in a consistent pattern between the diverse gender and sexual orientation group and cisgender and heterosexual group. However, compared to the full metric model, the full scalar invariance model was significantly different (*p* <.001). To account for differences in intercepts between groups, a partially invariant scalar model was constructed.

After investigating which variables would reduce the chi-square statistic in the partial scalar invariance model, the following intercepts were freed: Q14e, Q13d, and Q13e. In this case, 8% of items in the survey required free intercept scores (3/38). After freeing three intercepts in the partially invariant scalar model, it was no longer significantly different from the full metric model (*p* =.05). Additionally, the model fit statistics were similar across all versions of the three-factor model. However, while fit statistics met the criteria for the CF1 (≥ 0.90) and SRMR (≤ 0.08), they failed to meet the recommended cut-off for the RMSEA (≤ 0.06). A potential reason for the reduction in model fit statistics may have been the smaller sample size that was used to estimate the model (*n* = 322), especially given the large number of model parameters involved. Despite some degradation in model fit statistics, measurement invariance tests suggest that the TIMHSS can be reliably administered to diverse populations of gender identities and sexual orientations.

Using the factor scores obtained from the partially invariant scalar model, a linear regression was performed on global health care trust scores using diverse gender and sexual orientation as a predictor variable. Compared to the cisgender and heterosexual reference group, a significant decrease in global health care trust was observed for those in the diverse gender and sexual orientation group (β=-0.63, SE = 0.22, *t*=-2.91, *p* =.004).

#### TIMHSS

Based on the review of existing measures, analysis of 72 qualitative interviews, an expert review consensus process, factor analyses and validation testing, we present a 38-item correlated three-factor model scale measuring trust in doctors, policies, and the system. The final survey and instructions for sure are available to readers upon request of the corresponding author.

## Discussion

The aim of the present research was to develop a measure of trust in healthcare. Although model fit statistics were identified as being slightly better for the unidimensional and two-factor models, the three-factor model demonstrated acceptable model fit, required fewer covarying residual error terms and crucially, had greater theoretical merit in predicting outcomes. Measurement of invariance models created for women and individuals of diverse gender identities and sexual orientations provide evidence to suggest that healthcare trust scores can also be derived directly from the three-factor model and compared across these groups.

Results from the validation study demonstrated convergent validity when looking at associations between the TIMHSS and measures of satisfaction and the Wake Forest Physician Trust Scale [37]. In terms of discriminant validity, weak associations were identified between the TIMHSS and not questioning medical advice, and a negligible relationship with feeling as though there is no choice but to follow a doctor’s recommendation. These data support our hypothesis that our measure is distinct from measures of dependence in health care.

With regards to criterion validity, global factor scores on the TIMHSS significantly predicted each of the criterion validity questions included in the study. The direction of effect for all outcome variables was consistent with a priori hypotheses; that is, individuals who trust tended to concord more with medication or treatment plans, were more willing to accept new vaccines and to disclose medically relevant information, and were less likely to delay access to care and request a second opinion. The measure is thus consistently predictive of theoretically relevant outcomes, demonstrating support for criterion validity.

More substantive analyses of the data from qualitative interviews regarding differences in trust between equity-deserving groups are presented elsewhere (e.g [23, 82–84]). However, the present analysis demonstrates that members of equity-deserving populations have the lowest levels of trust in cases where trust varied between demographic groups. As such, our work further supports the notion that members of these communities are less likely to trust healthcare, as a social institution. The System and Policy factors, and specifically items related to judgement, equity, advocacy, and mutual trust, allow us to identify systemic explanations for lower levels of trust among equity-deserving populations. It may be that trust is lower because Canadian social institutions have been structured in a way that advantages those already in a place of privilege (e.g [85]). This points to the continued need to better understand, measure, and respond to a lack of trust in these populations within and outside of Canada, and the importance of valid measures that permit such investigations.

Individual identities interact, intersect, and compound to shape systemic oppressions/privileges [86] that impact lived experiences with healthcare and will ultimately influence why one does, or does not, trust. We suggest future analyses be conducted to identify how and in what way(s) trust differs - that is, what are the items within the survey that have the greatest impact for diverse populations - through a lens of intersectionality. This will support a better understanding of why healthcare institutions are not considered trustworthy [82, 87], thus placing responsibility on doctors and the health system to demonstrate trustworthiness, rather than blaming patients for a lack of trust [9]. Data collected using valid measures of trust that look to how specific attributes related to the perceived trustworthiness of individuals and organisations influence trust can support initiatives that attempt to redress their failures in meeting the needs of these populations in the past and present. Our initial exploration of measurement invariance was demonstrated for women and members of diverse sexual orientations, indicating that with some adjustments to estimation of model parameters, trust scores can be reliably compared among these subgroups. However, factor scores need to be generated to make accurate comparisons, adding greater complexity to the sample size and analytic code required to analyse data from the TIMHSS.

### Future research

Within the present study we did not aim to provide an overall score for the scale, meaning that researchers/healthcare organisations using the scale for measurement and evaluation purposes may need to generate an agreed upon index or scoring that is relevant in the given context. We intentionally chose not to recommend how scores should be interpreted because we are measuring a social construct and the meaning ascribed to the scores will differ based on the research question (and approach to analysis) or in the case of its practical use, based on the organisation/population of focus. Organisations or researchers interested in using the measure are welcome to contact the corresponding author to discuss conceptual or practical considerations for scoring in specific clinical or research contexts. Creating a smaller survey with a subset of items is also a recommended next step to reduce the time burden for respondents and analysts that stem from a 38-item scale. Considerations for shortening the scale include using more stringent cut-off criteria for factor cross-loadings and communality scores, as well as measurement variance between demographic groups. We also suggest that items 13b and 13c be revisited in future. In the present analysis, these items (‘I trust that the privatization of health care services does not disadvantage patients’, ‘I trust that healthcare will be affordable for me’), were removed due to uniqueness and poor factor loading scores (along with 13 g, 14a, and 15b). 13b and 13c items were included given political shifts in Canada at the time of data collection to increase for-profit private services, positioned by leaders as a response to an overwhelmed public system due to COVID-19. We expect these items to have greater relevance in future within the Canadian context though their relevance internationally will vary somewhat. Ongoing research into measurement invariance and comparisons of trust scores among various equity-deserving groups should also be considered in future work. This will allow for a more precise validation of the TIMHSS and enhance our understanding of the structural and procedural elements of healthcare that underserve these populations. Finally, we recommend researchers conduct replication studies to work towards validating our measure.

### Limitations

We acknowledge that this work was conducted in Canada and as a result, while the measure is likely applicable across cultures, modifications will be needed to take emic constructs and local practices into account [21]. We also acknowledge that the ‘dependence’ construct used for discriminant validity is based on limited research. Additionally, the item used to measure dependence, as well as three additional items used for criterion and convergent validity, were developed by our research team given the absence of an existing measure, which may be considered a limitation in our validation study. Finally, the use of the word ‘trust’ in our measure may be considered a methodological concern. Henwood et al. [88], for example, suggest that the use of a construct within a research tool designed to interrogate the same construct might impose a particular frame of reference and influence participant responses. We acknowledge that participant responses might vary if scale items did not introduce the word trust. As a future methodological exercise, we suggest a research design that involves adjusting the scale, collecting data using both the original and adjusted measures, and comparing responses.

## Concluding remarks

Our work was designed to respond to noted limitations of respected yet dated measures of trust in healthcare. Following the conception of the present work, Richmond et al. [43] published three measures of trust based on a convenience sample (*N* = 801) of U.S. adults pre-pandemic: Trust in My Doctor (T-MD), Trust in Doctors in General (T-DiG), and Trust in the Health Care Team (T-HCT). Given the date of publication, these measures did not inform our study design, but we acknowledge their substantial contribution to the literature, particularly within the American context where private health insurance may have greater relevance to trust in healthcare. We also feel it critical to highlight our novel contributions, distinct from Richmond et al., with regards to time of measurement development (pre- vs. post-pandemic), sample (one dataset vs. two separate waves for EFA and CFA; oversampling of equity-deserving populations), conceptual content (e.g., including dependence as a measure for criterion validity) and thus, application. In detail, we drew on well-established scales and created/removed/adjusted items based on data generated largely post-COVID and following major social movements (e.g., in response to the murder of George Floyd) that changed discussions regarding social institutions, including how healthcare should operate, globally. Relatedly, we are the first team to oversample multiple sub-populations historically disadvantaged by social institutions that might provide more insight into the concept of trust, leading to the creation of items reflecting judgement (‘I trust that doctors do not judge their patients’), equity (‘I trust that doctors treat all patients the same’), advocacy (‘I trust that doctors will always stick up for their patients’) and mutual trust (‘I trust that doctors trust me’). The last item, mutual trust, has been identified as a novel area of investigation – that is, examining the ways in which patient trust in clinician is influenced by clinician trust in patients – as social scientists and policymakers tend to focus more on individual factors and behaviours rather than traits of patient–physician pairs [9]. We are also the first to respond to conceptual challenges in the measurement of trust as distinct from dependence. Finally, in terms of comprehensiveness, while others have developed individual measures that focus on the system or individual, we are the first to create one measure that examines trust in healthcare broadly, including doctor, system, and policy - yielding a better global measure of trust in healthcare.

The COVID-19 pandemic has reignited a commitment to rebuilding trust [9] and an appetite for measures required to keep a ‘finger on the pulse’ of trust [89]. Our measure should be used to track trust over time at a population level, or within specific subpopulations, to identify and inform interventions to build trust. It is only once we understand the dimensions and related items of trust that are problematic that we might design means for (re)building trust. Our measure should also be used in interventions studies within a clinical setting to look at associations between trust and patient outcomes, as the evidence base for this association remains short in supply due to a lack of intervention and experimental studies [9, 14].

The health policy and research community are paying greater attention than ever before to the importance of trust. The TIMHSS can and should be shared widely and adapted for current and future use in other jurisdictions beyond Canada to monitor and evaluate trust in healthcare and generate an evidence base for the association between trust and therapeutic outcomes.

### Electronic supplementary material

Below is the link to the electronic supplementary material.

**Supplementary Material 1: Additional file 1.** Correlation matrix plot (wave two survey data)

**Supplementary Material 2: Additional file 2.** Three-factor model parameters

## Data Availability

Data are not available as it is a requirement of the ethics committee that data remain confidential.
