# Supplementary material for: Development and validation of the Trust in Multidimensional Healthcare Systems Scale (TIMHSS)
Source: Int J Equity Health. 2024 May 8;23:94. doi: 10.1186/s12939-024-02162-y (PMC11078716; doi:10.1186/s12939-024-02162-y)
Supplement: Supplementary file 2 — Supplementary Material 2: Additional file 2. Three-factor model parameters [file 12939_2024_2162_MOESM2_ESM.docx]

Additional file 2: Three-factor model parameters.

| **Factor** | **Item** | **Estimate** | **SE** | **z-value** | **P** | **Loadings** |
| --- | --- | --- | --- | --- | --- | --- |
| Doctor | Q12b-I trust that doctors treat all patients the same | 1.00 | 0.89 | 0.77 |  |  |
|  | Q12a-I trust that doctors put patients’ interests ahead of their own | 0.71 | 0.04 | 19.85 | 0.00 | 0.67 |
|  | Q12c-I trust that doctors do not judge their patients | 1.03 | 0.03 | 34.35 | 0.00 | 0.78 |
|  | Q12d-I trust that doctors are responsive to feedback they receive from their patients | 0.94 | 0.04 | 26.86 | 0.00 | 0.81 |
|  | Q12e-I trust that doctors do not take advantage of their patients | 0.80 | 0.04 | 22.23 | 0.00 | 0.74 |
|  | Q12f-I trust that patients are taken seriously | 1.00 | 0.04 | 27.59 | 0.00 | 0.85 |
|  | Q12g-I trust patients get enough attention | 1.05 | 0.04 | 25.26 | 0.00 | 0.82 |
|  | Q12h-I trust that patients are listened to | 1.07 | 0.04 | 28.35 | 0.00 | 0.87 |
|  | Q12i-I trust that doctors spend enough time on their patients | 0.99 | 0.04 | 24.42 | 0.00 | 0.76 |
|  | Q12j-I trust that doctors will always stick up for their patients | 0.98 | 0.04 | 25.35 | 0.00 | 0.80 |
|  | Q12k-I trust that doctors can relate to their patients’ problems | 0.94 | 0.04 | 23.48 | 0.00 | 0.77 |
|  | Q12l-I trust that doctors will be consistent in the care they provide | 0.98 | 0.04 | 26.17 | 0.00 | 0.83 |
|  | Q12m-I trust that doctors trust me | 0.92 | 0.04 | 23.56 | 0.00 | 0.79 |
| System | Q13g-I trust that doctors have control over the decisions they make about my care | 1.00 | 0.63 | 0.60 |  |  |
|  | Q14a- I trust that doctors will admit when they have made mistakes | 1.11 | 0.07 | 15.19 | 0.00 | 0.61 |
|  | Q14b-I trust that doctors are committed to continuing their education and training | 0.93 | 0.06 | 15.41 | 0.00 | 0.64 |
|  | Q14c-I trust that doctors are knowledgeable about a range of diseases | 0.97 | 0.07 | 14.64 | 0.00 | 0.64 |
|  | Q14d-I trust that new treatments are put into practice in the healthcare system | 1.04 | 0.07 | 16.00 | 0.00 | 0.65 |
|  | Q14e-I trust that the education and training of doctors in this country is one of the world’s best | 0.98 | 0.07 | 14.44 | 0.00 | 0.62 |
|  | Q14f-I trust that doctors will continue to respond to new and emerging medical problems | 0.99 | 0.06 | 16.04 | 0.00 | 0.67 |
|  | Q15a-I trust that patients always get the right dose of medicine | 1.27 | 0.08 | 16.34 | 0.00 | 0.74 |
|  | Q15b-I trust that patients are referred to specialists in time | 1.32 | 0.08 | 16.57 | 0.00 | 0.71 |
|  | Q15c-I trust that patients always get the right type of medicine | 1.36 | 0.08 | 16.73 | 0.00 | 0.79 |
|  | Q15d- I trust that doctors will prescribe medicines at the appropriate time (not too early or too late) | 1.30 | 0.08 | 16.29 | 0.00 | 0.78 |
|  | Q15e-I trust that patients’ medical information is kept confidential | 1.09 | 0.08 | 14.44 | 0.00 | 0.64 |
|  | Q15f-I trust that doctors do enough tests (not too few or too many) | 1.35 | 0.08 | 16.58 | 0.00 | 0.79 |
|  | Q15g-I trust that patients will always get the best treatment | 1.46 | 0.08 | 17.73 | 0.00 | 0.85 |
|  | Q15h-I trust that doctors will make the right diagnosis | 1.28 | 0.07 | 17.63 | 0.00 | 0.82 |
|  | Q16a-I trust that the information given to patients is clear and understandable | 1.08 | 0.07 | 15.63 | 0.00 | 0.71 |
|  | Q16b-I trust that patients get sufficient information about the cause of their problems | 1.27 | 0.08 | 16.58 | 0.00 | 0.78 |
|  | Q16c-I trust that doctors discuss things fully with their patients | 1.31 | 0.08 | 16.07 | 0.00 | 0.77 |
|  | Q16d-I trust that patients get sufficient information about the various treatment options that are available | 1.34 | 0.08 | 17.20 | 0.00 | 0.79 |
|  | Q16e-I trust that patients get sufficient information about the effects of their treatment | 1.35 | 0.08 | 17.07 | 0.00 | 0.78 |
|  | Q16f-I trust that doctors make use of the patients’ own understanding and insights | 1.23 | 0.08 | 15.75 | 0.00 | 0.76 |
|  | Q17a-I trust that healthcare providers are good at cooperating with each other | 1.15 | 0.07 | 17.28 | 0.00 | 0.69 |
|  | Q17b-I trust that patients are not given conflicting information | 1.25 | 0.08 | 16.60 | 0.00 | 0.74 |
|  | Q17c-I trust that high levels of specialisation benefits the healthcare system | 0.87 | 0.06 | 14.00 | 0.00 | 0.58 |
| Policy | Q13a-I trust that the health system has the staffing and resources needed to provide the care Canadians need | 1.00 | 0.89 | 0.71 |  |  |
|  | Q13d-I trust that medical help and patient care will not be compromised by waiting lists | 1.18 | 0.05 | 22.38 | 0.00 | 0.86 |
|  | Q13e-I trust that patients will not be the victims of the rising costs of health care | 1.07 | 0.05 | 20.67 | 0.00 | 0.79 |
|  | Q13f-I trust that waiting times are never too long | 1.13 | 0.05 | 21.05 | 0.00 | 0.83 |
| Covariances | .Q14a | ~~ |  |  |  |  |
|  | .Q14b | 0.14 | 0.03 | 5.45 | 0.00 | 0.22 |
|  | .Q14b | ~~ |  |  |  |  |
|  | Q14c-I trust that doctors are knowledgeable about a range of diseases | 0.21 | 0.03 | 8.30 | 0.00 | 0.40 |
|  | Q14d-I trust that new treatments are put into practice in the healthcare system | 0.14 | 0.03 | 5.26 | 0.00 | 0.25 |
|  | Q14e-I trust that the education and training of doctors in this country is one of the world’s best | 0.17 | 0.03 | 6.60 | 0.00 | 0.31 |
|  | Q14f-I trust that doctors will continue to respond to new and emerging medical problems | 0.21 | 0.02 | 9.10 | 0.00 | 0.44 |
|  | Q14c | ~~ |  |  |  |  |
|  | Q14d-I trust that new treatments are put into practice in the healthcare system | 0.18 | 0.03 | 6.78 | 0.00 | 0.33 |
|  | Q14e-I trust that the education and training of doctors in this country is one of the world’s best | 0.18 | 0.03 | 6.32 | 0.00 | 0.32 |
|  | Q14f-I trust that doctors will continue to respond to new and emerging medical problems | 0.23 | 0.02 | 9.57 | 0.00 | 0.47 |
|  | Q14d | ~~ |  |  |  |  |
|  | Q14e-I trust that the education and training of doctors in this country is one of the world’s best | 0.20 | 0.03 | 6.20 | 0.00 | 0.34 |
|  | Q14f-I trust that doctors will continue to respond to new and emerging medical problems | 0.21 | 0.03 | 8.11 | 0.00 | 0.40 |
|  | Q14e | ~~ |  |  |  |  |
|  | Q14f-I trust that doctors will continue to respond to new and emerging medical problems | 0.27 | 0.03 | 10.19 | 0.00 | 0.50 |
|  | Q15a | ~~ |  |  |  |  |
|  | Q15b-I trust that patients are referred to specialists in time | 0.14 | 0.03 | 4.54 | 0.00 | 0.22 |
|  | Q15b | ~~ |  |  |  |  |
|  | Q15c-I trust that patients always get the right type of medicine | 0.14 | 0.03 | 5.34 | 0.00 | 0.26 |
|  | Q15a | ~~ |  |  |  |  |
|  | Q15c-I trust that patients always get the right type of medicine | 0.17 | 0.03 | 6.36 | 0.00 | 0.34 |
|  | Q15c | ~~ |  |  |  |  |
|  | Q15d- I trust that doctors will prescribe medicines at the appropriate time (not too early or too late) | 0.11 | 0.02 | 5.12 | 0.00 | 0.24 |
|  | Q16a | ~~ |  |  |  |  |
|  | Q16b-I trust that patients get sufficient information about the cause of their problems | 0.14 | 0.02 | 6.38 | 0.00 | 0.33 |
|  | Q16b | ~~ |  |  |  |  |
|  | Q16c-I trust that doctors discuss things fully with their patients | 0.13 | 0.02 | 6.19 | 0.00 | 0.29 |
|  | Q16c | ~~ |  |  |  |  |
|  | Q16d-I trust that patients get sufficient information about the various treatment options that are available | 0.17 | 0.02 | 7.30 | 0.00 | 0.37 |
|  | Q16e-I trust that patients get sufficient information about the effects of their treatment | 0.12 | 0.02 | 5.27 | 0.00 | 0.26 |
|  | Q16d | ~~ |  |  |  |  |
|  | Q16e-I trust that patients get sufficient information about the effects of their treatment | 0.21 | 0.03 | 7.16 | 0.00 | 0.46 |
|  | Q17a | ~~ |  |  |  |  |
|  | Q17b-I trust that patients are not given conflicting information | 0.19 | 0.03 | 6.73 | 0.00 | 0.34 |
|  | Doctor | ~~ |  |  |  |  |
|  | System | 0.46 | 0.04 | 12.12 | 0.00 | 0.82 |
|  | Policy | 0.38 | 0.04 | 9.75 | 0.00 | 0.49 |
|  | System | ~~ |  |  |  |  |
|  | Policy | 0.33 | 0.04 | 9.20 | 0.00 | 0.59 |

Note: The '~~' beside a question denotes that error terms for the following questions are covarying with them. For example, for Q14b, its error terms were set to covary with those of 14c, 14d, 14e, and 14f.
