# Supplementary material for: Development and validation of the Trust in Multidimensional Healthcare Systems Scale (TIMHSS)
Source: Int J Equity Health. 2024 May 8;23:94. doi: 10.1186/s12939-024-02162-y (PMC11078716; doi:10.1186/s12939-024-02162-y)
Supplement: Supplementary file 1 — Supplementary Material 1: Additional file 1. Correlation matrix plot (wave two survey data) [file 12939_2024_2162_MOESM1_ESM.docx]

Additional file 1: Correlation matrix plot (wave two survey data).
